# Rubidium(I) monensinate dihydrate

**DOI:** 10.1107/S1600536807065221

**Published:** 2007-12-12

**Authors:** Sema Öztürk Yıldırım, Vickie McKee, Fatim-Zahra Khardli, Mostafa Mimouni, Taibi Ben Hadda

**Affiliations:** a Department of Physics, Faculty of Arts and Sciences, Erciyes University, 38039 Kayseri, Turkey; b Department of Chemistry, Loughborough University, Leicestershire LE11 3TU, England; c Laboratoire d’Activation Moléculaire, Faculté des Sciences, 60000 Oujda, Morocco

## Abstract

In the title complex, [Rb(C_36_H_61_O_11_)]·2H_2_O, the Rb^+^ cation is coordinated by seven O atoms of monensin. Rb—O distances range from 2.7870 (17) to 3.1429 (17) Å. Both O atoms of the carboxyl­ate group are involved in the coordination of Rb. The structure displays inter- and intra­molecular O—H⋯O and C—H⋯O hydrogen-bonding inter­actions.

## Related literature

For the crystal structures of some metal and alkali-metal complexes of monensin, see: Agtarap *et al.* (1967 ▶); Pinkerton & Steinrauf (1970 ▶); Walba *et al.* (1986 ▶); Barrans *et al.* (1982 ▶); Pangborn *et al.* (1987 ▶). For related literature, see: Euler *et al.* (2000 ▶); Grinstein *et al.* (1989 ▶); Mollenhauer *et al.* (1990 ▶); Pressman (1976 ▶); Singh *et al.* (2006 ▶); Westley (1983 ▶); Zhu & Loh (1995 ▶).
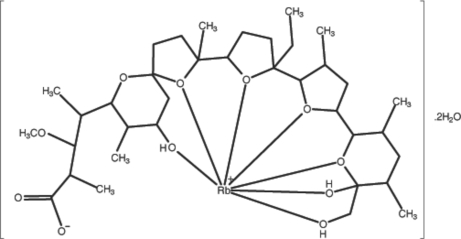


## Experimental

### 

#### Crystal data

[Rb(C_36_H_61_O_11_)]·2H_2_O
*M* *_r_* = 791.35
Orthorhombic, 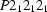

*a* = 12.5298 (15) Å
*b* = 16.361 (2) Å
*c* = 19.342 (2) Å
*V* = 3965.1 (8) Å^3^
*Z* = 4
Mo *K*α radiation
μ = 1.31 mm^−1^
*T* = 149 (2) K
0.32 × 0.26 × 0.26 mm

#### Data collection

Bruker SMART APEXII CCD area-detector diffractometer
Absorption correction: multi-scan (*SADABS*; Sheldrick, 2003 ▶) *T*_min_ = 0.679, *T*_max_ = 0.727
39930 measured reflections
9855 independent reflections
8673 reflections with *I* > 2σ(*I*)
*R*_int_ = 0.054

#### Refinement

*R*[*F* ^2^ > 2σ(*F* ^2^)] = 0.036
*wR*(*F* ^2^) = 0.087
*S* = 1.01
9855 reflections
479 parameters
H atoms treated by a mixture of independent and constrained refinement
Δρ_max_ = 0.69 e Å^−3^
Δρ_min_ = −0.42 e Å^−3^
Absolute structure: Flack (1983 ▶), 4380 Freidel pairs
Flack parameter: −0.011 (4)

### 

Data collection: *APEX2* (Bruker, 2005 ▶); cell refinement: *APEX2*; data reduction: *SAINT* (Bruker, 2005 ▶); program(s) used to solve structure: *SIR97* (Altomare *et al.*, 1999 ▶); program(s) used to refine structure: *SHELXL97* (Sheldrick, 1997 ▶); molecular graphics: *ORTEP-3 for Windows* (Farrugia, 1997 ▶); software used to prepare material for publication: *WinGX* (Farrugia, 1999 ▶).

## Supplementary Material

Crystal structure: contains datablocks global, I. DOI: 10.1107/S1600536807065221/bt2658sup1.cif

Structure factors: contains datablocks I. DOI: 10.1107/S1600536807065221/bt2658Isup2.hkl

Additional supplementary materials:  crystallographic information; 3D view; checkCIF report

## Figures and Tables

**Table 1:** Selected bond lengths (Å)

Rb1—O1	3.1429 (17)
Rb1—O2	2.9182 (19)
Rb1—O3	2.9125 (15)
Rb1—O4	2.8178 (16)
Rb1—O5	2.8679 (16)
Rb1—O6	2.7993 (16)
Rb1—O8	2.7870 (17)

**Table 2:** Hydrogen-bond geometry (Å, °)

*D*—H⋯*A*	*D*—H	H⋯*A*	*D*⋯*A*	*D*—H⋯*A*
O12—H1*WA*⋯O9^i^	0.78 (4)	2.20 (4)	2.959 (3)	167 (3)
O1—H1*O*⋯O11	0.86 (4)	1.82 (4)	2.651 (2)	162 (3)
O12—H1*WB*⋯O2	0.71 (3)	2.06 (3)	2.743 (3)	162 (4)
O2—H2*O*⋯O10	0.82 (4)	1.74 (4)	2.538 (3)	162 (3)
O13—H2*WA*⋯O1	0.92 (3)	1.93 (3)	2.807 (3)	158 (3)
O13—H2*WB*⋯O12^ii^	0.83 (4)	1.94 (4)	2.762 (3)	174 (3)
O8—H8*O*⋯O13	0.70 (3)	2.04 (3)	2.723 (3)	168 (3)
C10—H10*B*⋯O3	0.97	2.52	2.920 (3)	104
C17—H17*A*⋯O4	0.97	2.46	2.847 (3)	104
C18—H18*A*⋯O6	0.97	2.58	2.963 (3)	103
C21—H21*B*⋯O5	0.96	2.53	2.869 (3)	101
C29—H29⋯O6	0.98	2.60	2.924 (3)	100
C31—H31*C*⋯O7	0.96	2.44	2.787 (3)	101
C35—H35*A*⋯O9	0.96	2.43	2.813 (3)	103

Symmetry codes: (i) 
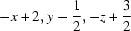
; (ii) 
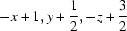
.

**Fig. 1.**
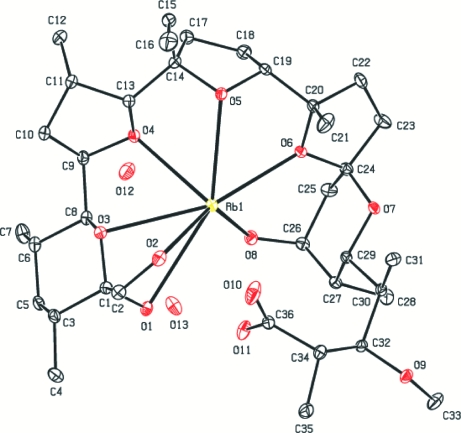
ORTEP view of the title compound with the atom numbering scheme. Displacement ellipsoids for non-H atoms are drawn at the 20% probability level. For clarity, H atoms have been omitted.

## supplementary crystallographic information

### Comment

Monensin is a carboxyl polyether ionophore produced by *Streptomyces cinnamonensin *(Mollenhauer *et al.*, 1990). It has been known for many years in poultry industry for its useful effect as food additive. It is also well known as a Na^+^/H^+^ exchanger across biological and model membranes. Being an ionophoric antibiotic, monensin is able to form lipophilic complexes with monovalent cations, hence, can cause cation imbalances which are known to produce different biochemical and histological changes (Mollenhauer *et al.*, 1990). We report here the synthesis and structure of a rubidium(I)-monensin complex (Fig. 1.).

The rubidium is sevenfold-coordinated *via* seven O atoms with Rb—O distances in the range from 2.7870 (17) Å to 3.1429 (17) Å. The oxygen atoms of the two water molecules do not coordinated to the Rb^+^ cation. The Rb—O distances compares with those in the range 3.06 - 3.08 reported by Euler *et al.* (2000).

The crystal structure is stabilized by inter- and intramolecular O—H···O and C—H···O hydrogen bonding interactions (Table 2).

### Experimental

A mixture of monensin acid (500 mg, 0.75 mmol), and RbOCH_3_ (87.4 mg, 0.75 mmol) in methanol was stirred for 20 min. After this time, the solvent was evaporated under reduced pressure to dryness. The residue was dissolved in dried acetonitrile/toluene (*v*/*v*:1/2). The solution was allowed to evaporate at room temperature. After one week, crystals suitable for X-ray diffraction were obtained by recrystallization from a mixture of dried acetonitrile / toluene (*v*/*v*:1/:2).

### Refinement

The H atoms of the two water molecules and hydroxyl groups were located in a difference Fourier map and freely refined. All other H atoms were geometrically positioned and treated as riding on their parent atoms, with C—H = 0.96 (CH_3_), 0.97 (CH_2_) or 0.98 Å(CH), and with *U*_iso_(H) = 1.5*U*_eq_(CH_3_) or 1.2*U*_eq_(CH_2_,CH).

### Crystal data

**Table:**

[Rb(C_36_H_61_O_11_)]·2H_2_O	*F*_000_ = 1688
*M**_r_* = 791.35	*D*_x_ = 1.326 Mg m^−^^3^
Orthorhombic, *P*2_1_2_1_2_1_	Mo *K*α radiation λ = 0.71069 Å
Hall symbol: P 2ac 2ab	Cell parameters from 9933 reflections
*a* = 12.5298 (15) Å	θ = 2.5–27.4º
*b* = 16.361 (2) Å	µ = 1.31 mm^−^^1^
*c* = 19.342 (2) Å	*T* = 149 (2) K
*V* = 3965.1 (8) Å^3^	Wedge, colourless
*Z* = 4	0.32 × 0.26 × 0.26 mm

### Data collection

**Table:**

Bruker SMART APEXII CCD area-detector diffractometer	9855 independent reflections
Radiation source: sealed tube	8673 reflections with *I* > 2σ(*I*)
Monochromator: graphite	*R*_int_ = 0.054
*T* = 149(2) K	θ_max_ = 28.4º
φ and ω scans	θ_min_ = 1.6º
Absorption correction: multi-scan(SADABS; Sheldrick, 2003)	*h* = −16→16
*T*_min_ = 0.679, *T*_max_ = 0.727	*k* = −21→21
39930 measured reflections	*l* = −25→25

### Refinement

**Table:**

Refinement on *F*^2^	Hydrogen site location: inferred from neighbouring sites
Least-squares matrix: full	H atoms treated by a mixture of independent and constrained refinement
*R*[*F*^2^ > 2σ(*F*^2^)] = 0.036	*w* = 1/[σ^2^(*F*_o_^2^) + (0.0519*P*)^2^] where *P* = (*F*_o_^2^ + 2*F*_c_^2^)/3
*wR*(*F*^2^) = 0.087	(Δ/σ)_max_ = 0.002
*S* = 1.01	Δρ_max_ = 0.69 e Å^−^^3^
9855 reflections	Δρ_min_ = −0.41 e Å^−^^3^
479 parameters	Extinction correction: none
Primary atom site location: structure-invariant direct methods	Absolute structure: Flack (1983), 4380 Freidel pairs
Secondary atom site location: difference Fourier map	Flack parameter: −0.011 (4)

### Special details

**Table:**

Geometry. Bond distances, angles *etc*. have been calculated using the rounded fractional coordinates. All su's are estimated from the variances of the (full) variance-covariance matrix. The cell e.s.d.'s are taken into account in the estimation of distances, angles and torsion angles
Refinement. Refinement on *F*^2^ for ALL reflections except those flagged by the user for potential systematic errors. Weighted *R*-factors *wR* and all goodnesses of fit *S* are based on *F*^2^, conventional *R*-factors *R* are based on *F*, with *F* set to zero for negative *F*^2^. The observed criterion of *F*^2^ > σ(*F*^2^) is used only for calculating -*R*-factor-obs *etc*. and is not relevant to the choice of reflections for refinement. *R*-factors based on *F*^2^ are statistically about twice as large as those based on *F*, and *R*-factors based on ALL data will be even larger.

### Fractional atomic coordinates and isotropic or equivalent isotropic displacement parameters (Å^2^)

**Table:**

	*x*	*y*	*z*	*U*_iso_*/*U*_eq_	
Rb1	0.68058 (2)	0.62540 (1)	0.83254 (1)	0.0275 (1)	
O1	0.55574 (13)	0.68922 (9)	0.70235 (9)	0.0251 (5)	
O2	0.71406 (13)	0.54905 (10)	0.69792 (10)	0.0308 (5)	
O3	0.51155 (11)	0.56050 (9)	0.74496 (8)	0.0223 (4)	
O4	0.50828 (12)	0.53649 (9)	0.88830 (8)	0.0259 (4)	
O5	0.71541 (12)	0.51248 (9)	0.94368 (8)	0.0242 (4)	
O6	0.76779 (12)	0.68235 (9)	0.95677 (8)	0.0239 (4)	
O7	0.88292 (12)	0.79223 (9)	0.93511 (8)	0.0236 (4)	
O8	0.61547 (14)	0.78001 (10)	0.87589 (10)	0.0302 (5)	
O9	1.03489 (12)	0.90146 (9)	0.73048 (9)	0.0295 (5)	
O10	0.86292 (17)	0.65349 (12)	0.70870 (15)	0.0650 (8)	
O11	0.74847 (14)	0.75533 (11)	0.71053 (10)	0.0382 (6)	
C1	0.53415 (17)	0.60717 (11)	0.68444 (11)	0.0226 (6)	
C2	0.63005 (19)	0.56494 (13)	0.65155 (12)	0.0281 (7)	
C3	0.43701 (19)	0.60519 (13)	0.63593 (12)	0.0279 (7)	
C4	0.4544 (2)	0.65092 (17)	0.56827 (14)	0.0429 (8)	
C5	0.33877 (17)	0.63550 (13)	0.67509 (12)	0.0291 (6)	
C6	0.31882 (19)	0.58736 (13)	0.74052 (12)	0.0280 (6)	
C7	0.22450 (19)	0.62327 (19)	0.77985 (15)	0.0419 (8)	
C8	0.42073 (16)	0.58663 (13)	0.78455 (12)	0.0227 (6)	
C9	0.41327 (17)	0.52918 (13)	0.84666 (12)	0.0266 (7)	
C10	0.40360 (18)	0.43782 (13)	0.82942 (14)	0.0302 (6)	
C11	0.4651 (2)	0.39624 (14)	0.88795 (13)	0.0320 (7)	
C12	0.3948 (2)	0.38367 (19)	0.95158 (14)	0.0473 (9)	
C13	0.55770 (18)	0.45711 (13)	0.89552 (12)	0.0258 (6)	
C14	0.62747 (18)	0.45782 (13)	0.96051 (12)	0.0271 (6)	
C15	0.6740 (2)	0.37246 (15)	0.97546 (14)	0.0400 (8)	
C16	0.7465 (3)	0.33811 (18)	0.92008 (19)	0.0537 (10)	
C17	0.5754 (2)	0.49580 (16)	1.02467 (13)	0.0343 (8)	
C18	0.6482 (2)	0.56660 (15)	1.04553 (13)	0.0348 (7)	
C19	0.75078 (19)	0.54845 (13)	1.00711 (12)	0.0260 (6)	
C20	0.82715 (17)	0.61938 (13)	0.99252 (12)	0.0288 (6)	
C21	0.9200 (2)	0.59105 (17)	0.94769 (19)	0.0488 (9)	
C22	0.8648 (3)	0.65952 (16)	1.05960 (16)	0.0471 (9)	
C23	0.8526 (2)	0.74925 (15)	1.04981 (13)	0.0388 (8)	
C24	0.80195 (18)	0.76179 (13)	0.97872 (11)	0.0254 (6)	
C25	0.7057 (2)	0.81916 (15)	0.97967 (12)	0.0315 (7)	
C26	0.66921 (17)	0.84601 (13)	0.90796 (12)	0.0251 (6)	
C27	0.76439 (15)	0.87581 (13)	0.86451 (11)	0.0230 (5)	
C28	0.80635 (19)	0.95721 (14)	0.89261 (14)	0.0331 (7)	
C29	0.85077 (16)	0.80908 (12)	0.86470 (11)	0.0211 (6)	
C30	0.95440 (16)	0.83083 (12)	0.82654 (12)	0.0229 (6)	
C31	1.03786 (17)	0.76331 (14)	0.83467 (14)	0.0310 (7)	
C32	0.94094 (16)	0.85553 (12)	0.75006 (12)	0.0236 (6)	
C33	1.0276 (2)	0.98639 (15)	0.74268 (17)	0.0426 (9)	
C34	0.93353 (18)	0.78700 (14)	0.69649 (13)	0.0286 (7)	
C35	0.9230 (2)	0.82303 (18)	0.62323 (14)	0.0415 (9)	
C36	0.84047 (18)	0.72763 (14)	0.70674 (12)	0.0275 (7)	
O12	0.73374 (18)	0.38982 (14)	0.74038 (13)	0.0476 (8)	
O13	0.45804 (17)	0.81107 (12)	0.78250 (10)	0.0396 (6)	
H1O	0.622 (3)	0.7020 (17)	0.6994 (16)	0.044 (8)*	
H2A	0.60670	0.51370	0.63150	0.0340*	
H2B	0.65680	0.59900	0.61430	0.0340*	
H2O	0.758 (3)	0.586 (2)	0.6933 (17)	0.052 (10)*	
H3	0.42410	0.54780	0.62410	0.0330*	
H4A	0.51640	0.62950	0.54530	0.0640*	
H4B	0.46490	0.70800	0.57770	0.0640*	
H4C	0.39310	0.64410	0.53910	0.0640*	
H5A	0.34840	0.69270	0.68680	0.0350*	
H5B	0.27670	0.63130	0.64530	0.0350*	
H6	0.30150	0.53090	0.72780	0.0340*	
H7A	0.21250	0.59220	0.82120	0.0630*	
H7B	0.16190	0.62120	0.75130	0.0630*	
H7C	0.23960	0.67900	0.79180	0.0630*	
H8	0.43400	0.64220	0.80140	0.0270*	
H8O	0.578 (2)	0.7943 (17)	0.8520 (15)	0.031 (8)*	
H9	0.35160	0.54520	0.87470	0.0320*	
H10A	0.32950	0.42060	0.82900	0.0360*	
H10B	0.43540	0.42570	0.78490	0.0360*	
H11	0.49260	0.34340	0.87200	0.0380*	
H12A	0.43570	0.35780	0.98740	0.0710*	
H12B	0.33520	0.34970	0.93960	0.0710*	
H12C	0.36950	0.43560	0.96770	0.0710*	
H13	0.60510	0.44940	0.85580	0.0310*	
H15A	0.71370	0.37500	1.01850	0.0480*	
H15B	0.61530	0.33470	0.98230	0.0480*	
H16A	0.77150	0.28510	0.93400	0.0810*	
H16B	0.80630	0.37400	0.91360	0.0810*	
H16C	0.70770	0.33340	0.87750	0.0810*	
H17A	0.50430	0.51550	1.01400	0.0410*	
H17B	0.57040	0.45600	1.06170	0.0410*	
H18A	0.61830	0.61870	1.03130	0.0420*	
H18B	0.65980	0.56740	1.09510	0.0420*	
H19	0.79000	0.50670	1.03320	0.0310*	
H21A	0.96680	0.63630	0.93880	0.0730*	
H21B	0.89320	0.57020	0.90470	0.0730*	
H21C	0.95860	0.54880	0.97130	0.0730*	
H22A	0.82190	0.64100	1.09830	0.0570*	
H22B	0.93880	0.64580	1.06870	0.0570*	
H23A	0.92170	0.77600	1.05190	0.0470*	
H23B	0.80740	0.77200	1.08560	0.0470*	
H25A	0.64680	0.79190	1.00270	0.0380*	
H25B	0.72360	0.86730	1.00650	0.0380*	
H26	0.61880	0.89140	0.91320	0.0300*	
H27	0.74020	0.88420	0.81680	0.0280*	
H28A	0.86570	0.97510	0.86510	0.0500*	
H28B	0.82900	0.95020	0.93960	0.0500*	
H28C	0.75070	0.99740	0.89070	0.0500*	
H29	0.82100	0.75910	0.84450	0.0250*	
H30	0.98350	0.87890	0.85010	0.0270*	
H31A	1.10200	0.77880	0.81090	0.0460*	
H31B	1.01080	0.71340	0.81540	0.0460*	
H31C	1.05310	0.75540	0.88280	0.0460*	
H32	0.87810	0.89080	0.74570	0.0280*	
H33A	1.09270	1.01240	0.72850	0.0640*	
H33B	1.01620	0.99600	0.79110	0.0640*	
H33C	0.96900	1.00850	0.71680	0.0640*	
H34	1.00000	0.75550	0.69840	0.0340*	
H35A	0.98060	0.86050	0.61490	0.0620*	
H35B	0.85620	0.85140	0.61930	0.0620*	
H35C	0.92560	0.77970	0.58980	0.0620*	
H1WA	0.795 (3)	0.385 (2)	0.7462 (19)	0.059 (11)*	
H1WB	0.729 (3)	0.433 (2)	0.738 (2)	0.057 (12)*	
H2WA	0.478 (3)	0.776 (2)	0.7474 (18)	0.053 (9)*	
H2WB	0.401 (3)	0.834 (2)	0.7728 (18)	0.051 (9)*	

### Atomic displacement parameters (Å^2^)

**Table:**

	*U*^11^	*U*^22^	*U*^33^	*U*^12^	*U*^13^	*U*^23^
Rb1	0.0285 (1)	0.0310 (1)	0.0231 (1)	−0.0076 (1)	−0.0049 (1)	0.0030 (1)
O1	0.0241 (8)	0.0192 (7)	0.0319 (9)	−0.0007 (6)	0.0014 (7)	−0.0050 (6)
O2	0.0241 (8)	0.0246 (8)	0.0437 (10)	0.0021 (6)	0.0034 (7)	−0.0006 (7)
O3	0.0208 (7)	0.0226 (7)	0.0236 (8)	0.0022 (5)	0.0009 (6)	−0.0009 (6)
O4	0.0265 (8)	0.0252 (7)	0.0260 (8)	−0.0027 (6)	−0.0048 (7)	−0.0020 (6)
O5	0.0243 (7)	0.0269 (7)	0.0214 (8)	−0.0056 (6)	0.0004 (6)	−0.0014 (6)
O6	0.0243 (8)	0.0235 (7)	0.0239 (8)	−0.0007 (6)	−0.0054 (6)	0.0031 (6)
O7	0.0228 (7)	0.0244 (7)	0.0236 (8)	0.0011 (6)	−0.0073 (6)	0.0013 (6)
O8	0.0205 (8)	0.0322 (9)	0.0380 (10)	0.0009 (7)	−0.0049 (8)	0.0039 (7)
O9	0.0211 (7)	0.0299 (8)	0.0374 (10)	−0.0057 (6)	0.0018 (7)	0.0054 (7)
O10	0.0373 (11)	0.0296 (9)	0.128 (2)	−0.0031 (8)	−0.0201 (13)	0.0073 (12)
O11	0.0262 (8)	0.0324 (9)	0.0559 (12)	−0.0054 (7)	0.0093 (8)	−0.0108 (8)
C1	0.0270 (10)	0.0191 (10)	0.0216 (10)	0.0007 (7)	0.0006 (8)	−0.0028 (7)
C2	0.0322 (12)	0.0260 (10)	0.0260 (12)	0.0011 (9)	0.0066 (9)	−0.0067 (8)
C3	0.0334 (12)	0.0238 (11)	0.0265 (11)	−0.0017 (8)	−0.0076 (10)	−0.0038 (8)
C4	0.0530 (16)	0.0439 (14)	0.0317 (14)	−0.0035 (12)	−0.0150 (13)	0.0051 (11)
C5	0.0285 (11)	0.0257 (10)	0.0331 (12)	0.0006 (8)	−0.0134 (10)	−0.0007 (9)
C6	0.0223 (10)	0.0264 (10)	0.0352 (12)	−0.0006 (9)	−0.0032 (11)	−0.0055 (9)
C7	0.0237 (11)	0.0535 (15)	0.0486 (15)	0.0044 (12)	−0.0045 (11)	−0.0113 (14)
C8	0.0184 (9)	0.0216 (10)	0.0280 (11)	0.0006 (8)	0.0002 (9)	−0.0050 (8)
C9	0.0208 (10)	0.0316 (11)	0.0274 (13)	−0.0044 (8)	−0.0008 (9)	−0.0029 (9)
C10	0.0300 (11)	0.0281 (10)	0.0326 (12)	−0.0089 (8)	−0.0057 (11)	−0.0004 (10)
C11	0.0362 (12)	0.0273 (11)	0.0326 (13)	−0.0113 (9)	−0.0064 (11)	0.0023 (9)
C12	0.0438 (15)	0.0568 (17)	0.0414 (15)	−0.0259 (14)	−0.0036 (12)	0.0145 (14)
C13	0.0287 (11)	0.0247 (10)	0.0241 (11)	−0.0043 (9)	−0.0030 (9)	0.0005 (8)
C14	0.0281 (11)	0.0272 (11)	0.0260 (11)	−0.0075 (9)	0.0004 (9)	0.0026 (9)
C15	0.0478 (14)	0.0286 (11)	0.0437 (14)	−0.0091 (13)	−0.0167 (12)	0.0089 (11)
C16	0.0525 (18)	0.0327 (14)	0.076 (2)	0.0074 (13)	−0.0059 (17)	0.0012 (14)
C17	0.0327 (13)	0.0481 (14)	0.0220 (12)	−0.0100 (10)	0.0021 (10)	0.0009 (10)
C18	0.0443 (14)	0.0371 (12)	0.0231 (12)	−0.0081 (10)	0.0040 (10)	−0.0031 (10)
C19	0.0307 (11)	0.0249 (10)	0.0223 (11)	−0.0019 (8)	−0.0097 (9)	0.0033 (8)
C20	0.0239 (10)	0.0271 (10)	0.0355 (11)	−0.0015 (10)	−0.0118 (10)	0.0070 (9)
C21	0.0253 (12)	0.0362 (13)	0.085 (2)	0.0023 (10)	0.0074 (14)	0.0082 (14)
C22	0.0594 (18)	0.0330 (12)	0.0489 (17)	−0.0097 (12)	−0.0364 (15)	0.0071 (12)
C23	0.0549 (16)	0.0352 (12)	0.0264 (13)	0.0092 (11)	−0.0099 (12)	−0.0033 (10)
C24	0.0290 (12)	0.0255 (10)	0.0217 (10)	0.0011 (8)	−0.0014 (9)	−0.0018 (8)
C25	0.0366 (13)	0.0318 (11)	0.0262 (12)	0.0076 (9)	0.0043 (10)	0.0010 (9)
C26	0.0214 (10)	0.0283 (10)	0.0257 (11)	0.0063 (8)	0.0008 (9)	0.0010 (8)
C27	0.0200 (9)	0.0234 (9)	0.0257 (10)	0.0041 (9)	−0.0031 (8)	0.0030 (9)
C28	0.0300 (12)	0.0250 (10)	0.0444 (14)	0.0049 (9)	−0.0061 (11)	−0.0017 (10)
C29	0.0184 (9)	0.0210 (9)	0.0240 (11)	0.0012 (7)	−0.0034 (8)	0.0011 (8)
C30	0.0176 (9)	0.0240 (10)	0.0271 (11)	−0.0008 (7)	−0.0022 (9)	0.0016 (9)
C31	0.0220 (10)	0.0326 (11)	0.0383 (13)	0.0041 (8)	0.0035 (11)	0.0096 (11)
C32	0.0168 (9)	0.0261 (11)	0.0279 (11)	−0.0013 (7)	0.0006 (9)	0.0051 (8)
C33	0.0358 (14)	0.0265 (11)	0.0654 (19)	−0.0065 (10)	0.0033 (13)	0.0095 (12)
C34	0.0220 (11)	0.0309 (11)	0.0329 (13)	−0.0020 (9)	0.0036 (10)	0.0000 (9)
C35	0.0443 (15)	0.0494 (16)	0.0308 (14)	−0.0174 (12)	0.0044 (12)	0.0022 (12)
C36	0.0265 (12)	0.0302 (11)	0.0258 (12)	−0.0038 (8)	−0.0018 (9)	−0.0004 (9)
O12	0.0313 (11)	0.0356 (12)	0.0760 (16)	−0.0033 (8)	−0.0084 (10)	0.0165 (10)
O13	0.0387 (10)	0.0425 (10)	0.0375 (11)	0.0157 (8)	−0.0112 (9)	−0.0140 (8)

### Geometric parameters (Å, °)

**Table:**

Rb1—O1	3.1429 (17)	C32—C34	1.530 (3)
Rb1—O2	2.9182 (19)	C34—C35	1.540 (4)
Rb1—O3	2.9125 (15)	C34—C36	1.531 (3)
Rb1—O4	2.8178 (16)	C2—H2B	0.9700
Rb1—O5	2.8679 (16)	C2—H2A	0.9700
Rb1—O6	2.7993 (16)	C3—H3	0.9800
Rb1—O8	2.7870 (17)	C4—H4A	0.9600
O1—C1	1.413 (2)	C4—H4B	0.9600
O2—C2	1.407 (3)	C4—H4C	0.9600
O3—C1	1.426 (3)	C5—H5A	0.9700
O3—C8	1.437 (3)	C5—H5B	0.9700
O4—C9	1.442 (3)	C6—H6	0.9800
O4—C13	1.446 (3)	C7—H7B	0.9600
O5—C14	1.456 (3)	C7—H7C	0.9600
O5—C19	1.431 (3)	C7—H7A	0.9600
O6—C20	1.447 (3)	C8—H8	0.9800
O6—C24	1.433 (3)	C9—H9	0.9800
O7—C24	1.410 (3)	C10—H10B	0.9700
O7—C29	1.447 (3)	C10—H10A	0.9700
O8—C26	1.416 (3)	C11—H11	0.9800
O9—C32	1.447 (3)	C12—H12B	0.9600
O9—C33	1.412 (3)	C12—H12C	0.9600
O10—C36	1.246 (3)	C12—H12A	0.9600
O11—C36	1.241 (3)	C13—H13	0.9800
O1—H1O	0.86 (4)	C15—H15A	0.9700
O2—H2O	0.82 (4)	C15—H15B	0.9700
O8—H8O	0.70 (3)	C16—H16B	0.9600
O12—H1WA	0.78 (4)	C16—H16C	0.9600
O12—H1WB	0.71 (3)	C16—H16A	0.9600
O13—H2WB	0.83 (4)	C17—H17A	0.9700
O13—H2WA	0.92 (3)	C17—H17B	0.9700
C1—C2	1.525 (3)	C18—H18B	0.9700
C1—C3	1.537 (3)	C18—H18A	0.9700
C3—C4	1.523 (4)	C19—H19	0.9800
C3—C5	1.528 (3)	C21—H21B	0.9600
C5—C6	1.511 (3)	C21—H21C	0.9600
C6—C8	1.535 (3)	C21—H21A	0.9600
C6—C7	1.523 (4)	C22—H22A	0.9700
C8—C9	1.528 (3)	C22—H22B	0.9700
C9—C10	1.536 (3)	C23—H23A	0.9700
C10—C11	1.529 (4)	C23—H23B	0.9700
C11—C12	1.527 (4)	C25—H25A	0.9700
C11—C13	1.536 (3)	C25—H25B	0.9700
C13—C14	1.531 (3)	C26—H26	0.9800
C14—C15	1.541 (3)	C27—H27	0.9800
C14—C17	1.534 (3)	C28—H28A	0.9600
C15—C16	1.513 (4)	C28—H28B	0.9600
C17—C18	1.529 (4)	C28—H28C	0.9600
C18—C19	1.514 (3)	C29—H29	0.9800
C19—C20	1.530 (3)	C30—H30	0.9800
C20—C21	1.523 (4)	C31—H31B	0.9600
C20—C22	1.529 (4)	C31—H31C	0.9600
C22—C23	1.488 (4)	C31—H31A	0.9600
C23—C24	1.528 (3)	C32—H32	0.9800
C24—C25	1.528 (3)	C33—H33A	0.9600
C25—C26	1.525 (3)	C33—H33B	0.9600
C26—C27	1.538 (3)	C33—H33C	0.9600
C27—C28	1.532 (3)	C34—H34	0.9800
C27—C29	1.537 (3)	C35—H35B	0.9600
C29—C30	1.535 (3)	C35—H35C	0.9600
C30—C31	1.529 (3)	C35—H35A	0.9600
C30—C32	1.543 (3)		
			
Rb1···O10	3.342 (3)	H2WB···H1WB^v^	2.31 (5)
Rb1···O11	3.2881 (19)	H4A···C2	2.7100
Rb1···C29	3.737 (2)	H4A···H2B	2.2600
Rb1···C36	3.568 (2)	H4B···H11^v^	2.4800
Rb1···H21B	3.1400	H4B···O1	2.6800
Rb1···H29	2.8200	H4B···H5A	2.5800
Rb1···H33A^i^	3.5900	H4B···H15B^v^	2.5800
O1···O2	3.033 (2)	H4C···H5B	2.5300
O1···O11	2.651 (2)	H5A···H8	2.6000
O1···O13	2.807 (3)	H5A···O1	2.6200
O2···O12	2.743 (3)	H5A···H2WA	2.4200
O2···C36	3.328 (3)	H5A···H4B	2.5800
O2···O3	2.702 (2)	H5A···H7C	2.4600
O2···O10	2.538 (3)	H5B···H28C^iii^	2.3200
O2···O1	3.033 (2)	H5B···H4C	2.5300
O3···O2	2.702 (2)	H5B···H7B	2.5100
O3···C13	3.417 (3)	H6···H28C^iii^	2.4500
O3···O4	2.801 (2)	H6···C10	2.8000
O4···O5	2.835 (2)	H6···H3	2.5400
O4···O3	2.801 (2)	H6···C28^iii^	2.9500
O5···O6	2.867 (2)	H7A···H9	2.1700
O5···O4	2.835 (2)	H7A···C9	2.7600
O6···O8	2.940 (2)	H7B···H5B	2.5100
O6···O5	2.867 (2)	H7B···H33C^iii^	2.5400
O7···C21	3.333 (3)	H7C···C31^vii^	3.0000
O8···O13	2.723 (3)	H7C···H5A	2.4600
O8···O6	2.940 (2)	H7C···H8	2.5200
O9···O12^ii^	2.959 (3)	H7C···H31A^vii^	2.4000
O10···C33^i^	3.200 (3)	H8···O1	2.5700
O10···O2	2.538 (3)	H8···O13	2.8000
O10···Rb1	3.342 (3)	H8···H2WA	2.4900
O11···Rb1	3.2881 (19)	H8···H5A	2.6000
O11···C29	3.363 (3)	H8···H7C	2.5200
O11···O1	2.651 (2)	H8O···O13	2.04 (3)
O12···O2	2.743 (3)	H8O···H2WA	2.40 (5)
O12···C33^i^	3.398 (3)	H8O···H27	2.6000
O12···O9^i^	2.959 (3)	H8O···H22B^iv^	2.5200
O12···O13^iii^	2.762 (3)	H9···C7	2.7400
O13···O8	2.723 (3)	H9···C12	3.0800
O13···C22^iv^	3.305 (4)	H9···H7A	2.1700
O13···O12^v^	2.762 (3)	H9···H12C	2.5500
O13···O1	2.807 (3)	H10A···H12B	2.4300
O1···H2WA	1.93 (3)	H10B···O3	2.5200
O1···H5A	2.6200	H10B···H13	2.5600
O1···H8	2.5700	H10B···O13^iii^	2.6500
O1···H4B	2.6800	H11···C15	3.0700
O2···H1O	2.76 (3)	H11···H2WA^iii^	2.5900
O2···H33A^i^	2.8700	H11···H4B^iii^	2.4800
O2···H1WB	2.06 (3)	H12A···C14	2.9500
O3···H10B	2.5200	H12A···C15	3.0000
O4···H12C	2.8500	H12A···C17	2.9500
O4···H17A	2.4600	H12A···H15B	2.2800
O5···H21B	2.5300	H12B···H10A	2.4300
O5···H16B	2.6000	H12C···O4	2.8500
O6···H18A	2.5800	H12C···C17	2.9700
O6···H29	2.6000	H12C···H9	2.5500
O7···H31C	2.4400	H12C···C9	2.8500
O7···H21A	2.7600	H12C···H17A	2.3200
O7···H28B	2.6700	H13···H10B	2.5600
O8···H22B^iv^	2.7400	H13···H16C	2.3300
O8···H29	2.6700	H13···C16	2.8300
O9···H35A	2.4300	H15A···H17B	2.3800
O9···H1WA^ii^	2.20 (4)	H15A···H19	2.3700
O9···H31A	2.6700	H15A···C19	2.8800
O10···H2O	1.74 (4)	H15A···H2B^x^	2.5000
O10···H33A^i^	2.6700	H15B···H12A	2.2800
O11···H32	2.8300	H15B···H17B	2.5700
O11···H29	2.7500	H15B···C11	2.8100
O11···H35B	2.7200	H15B···C12	2.9400
O11···H2O	2.79 (3)	H15B···H4B^iii^	2.5800
O11···H1O	1.82 (4)	H16B···O5	2.6000
O12···H31A^i^	2.9200	H16C···O12	2.8300
O12···H16C	2.8300	H16C···H13	2.3300
O12···H2WB^iii^	1.94 (4)	H16C···C13	2.7800
O12···H22A^vi^	2.8800	H17A···C12	2.8300
O13···H8O	2.04 (3)	H17A···O4	2.4600
O13···H8	2.8000	H17A···H28B^iv^	2.4400
O13···H10B^v^	2.6500	H17A···H12C	2.3200
C7···C31^vii^	3.441 (4)	H17B···H15A	2.3800
C12···C15	3.533 (4)	H17B···H15B	2.5700
C12···C17	3.238 (4)	H18A···O6	2.5800
C15···C12	3.533 (4)	H18B···C22	3.0600
C17···C12	3.238 (4)	H18B···H22A	2.3600
C21···O7	3.333 (3)	H19···H22A	2.5600
C22···O13^viii^	3.305 (4)	H19···H21C	2.5200
C29···C36	3.336 (3)	H19···C15	2.8600
C29···O11	3.363 (3)	H19···H15A	2.3700
C29···Rb1	3.737 (2)	H19···C2^x^	2.7600
C31···C36	3.547 (3)	H19···H2A^x^	2.3200
C31···C7^ix^	3.441 (4)	H19···H2B^x^	2.4300
C33···O12^ii^	3.398 (3)	H21A···O7	2.7600
C33···O10^ii^	3.200 (3)	H21A···C24	3.0100
C36···C29	3.336 (3)	H21A···C31	3.0300
C36···C31	3.547 (3)	H21A···H22B	2.5400
C36···O2	3.328 (3)	H21A···H31C	2.4800
C36···Rb1	3.568 (2)	H21B···Rb1	3.1400
C1···H2WA	3.10 (3)	H21B···O5	2.5300
C2···H1WB	3.00 (4)	H21C···H19	2.5200
C2···H19^vi^	2.7600	H21C···H22B	2.4800
C2···H4A	2.7100	H22A···C18	2.6900
C3···H28C^iii^	2.9800	H22A···H18B	2.3600
C4···H2B	2.8200	H22A···H19	2.5600
C5···H28C^iii^	2.8300	H22A···O12^x^	2.8800
C6···H28C^iii^	3.0600	H22B···H21A	2.5400
C7···H9	2.7400	H22B···H21C	2.4800
C7···H31A^vii^	3.0300	H22B···O8^viii^	2.7400
C7···H33C^iii^	3.0700	H22B···C26^viii^	2.9300
C9···H12C	2.8500	H22B···H8O^viii^	2.5200
C9···H7A	2.7600	H22B···H26^viii^	2.3600
C10···H6	2.8000	H23B···H25A	2.5900
C11···H15B	2.8100	H23B···H25B	2.4200
C12···H15B	2.9400	H25A···H23B	2.5900
C12···H9	3.0800	H25B···C28	2.8400
C12···H17A	2.8300	H25B···H23B	2.4200
C13···H16C	2.7800	H25B···H28B	2.2900
C14···H12A	2.9500	H26···H28C	2.4300
C15···H19	2.8600	H26···H22B^iv^	2.3600
C15···H11	3.0700	H27···C32	2.8700
C15···H12A	3.0000	H27···H8O	2.6000
C16···H13	2.8300	H27···H32	2.2100
C17···H12C	2.9700	H28A···C30	2.7100
C17···H12A	2.9500	H28A···H30	2.1800
C18···H22A	2.6900	H28A···H33B	2.3900
C18···H30^iv^	3.0200	H28B···C25	2.7500
C19···H15A	2.8800	H28B···O7	2.6700
C22···H18B	3.0600	H28B···H25B	2.2900
C24···H21A	3.0100	H28B···H17A^viii^	2.4400
C25···H28B	2.7500	H28C···H5B^v^	2.3200
C26···H22B^iv^	2.9300	H28C···H26	2.4300
C27···H32	2.7100	H28C···C3^v^	2.9800
C28···H25B	2.8400	H28C···C5^v^	2.8300
C28···H6^v^	2.9500	H28C···C6^v^	3.0600
C28···H30	2.6900	H28C···H3^v^	2.3600
C30···H28A	2.7100	H28C···H6^v^	2.4500
C30···H33B	2.8900	H29···H31B	2.5600
C31···H21A	3.0300	H29···Rb1	2.8200
C31···H34	2.6800	H29···O6	2.6000
C31···H7C^ix^	3.0000	H29···O8	2.6700
C32···H27	2.8700	H29···O11	2.7500
C33···H30	2.7800	H29···C36	2.7200
C33···H1WA^ii^	2.78 (4)	H30···H28A	2.1800
C34···H31A	3.0600	H30···C28	2.6900
C34···H31B	2.7700	H30···C33	2.7800
C36···H31B	3.0000	H30···H33B	2.2700
C36···H29	2.7200	H30···C18^viii^	3.0200
C36···H1O	2.77 (4)	H31A···H34	2.5500
C36···H2O	2.55 (3)	H31A···O9	2.6700
H1WA···O9^i^	2.20 (4)	H31A···C7^ix^	3.0300
H1WA···H33A^i^	2.5600	H31A···C34	3.0600
H1WA···C33^i^	2.78 (4)	H31A···H7C^ix^	2.4000
H1WA···H31A^i^	2.4300	H31A···O12^ii^	2.9200
H1O···C36	2.77 (4)	H31A···H1WA^ii^	2.4300
H1O···O11	1.82 (4)	H31B···C34	2.7700
H1O···H2B	2.4000	H31B···C36	3.0000
H1O···H2O	2.55 (5)	H31B···H29	2.5600
H1O···H2WA	2.36 (5)	H31B···H34	2.3700
H1WB···C2	3.00 (4)	H31C···O7	2.4400
H1WB···H2WB^iii^	2.31 (5)	H31C···H21A	2.4800
H1WB···O2	2.06 (3)	H32···O11	2.8300
H2A···H3	2.3600	H32···C27	2.7100
H2A···H19^vi^	2.3200	H32···H27	2.2100
H2B···C4	2.8200	H32···H33B	2.5900
H2B···H1O	2.4000	H32···H33C	2.3100
H2B···H4A	2.2600	H32···H35B	2.5400
H2B···H15A^vi^	2.5000	H33A···Rb1^ii^	3.5900
H2B···H19^vi^	2.4300	H33A···O2^ii^	2.8700
H2O···C36	2.55 (3)	H33A···O10^ii^	2.6700
H2O···H1O	2.55 (5)	H33A···H1WA^ii^	2.5600
H2O···O10	1.74 (4)	H33B···C30	2.8900
H2O···O11	2.79 (3)	H33B···H28A	2.3900
H3···H2A	2.3600	H33B···H30	2.2700
H3···H28C^iii^	2.3600	H33B···H32	2.5900
H3···H6	2.5400	H33C···H32	2.3100
H2WA···C1	3.10 (3)	H33C···C7^v^	3.0700
H2WA···O1	1.93 (3)	H33C···H7B^v^	2.5400
H2WA···H8O	2.40 (5)	H34···C31	2.6800
H2WA···H11^v^	2.5900	H34···H31A	2.5500
H2WA···H1O	2.36 (5)	H34···H31B	2.3700
H2WA···H5A	2.4200	H35A···O9	2.4300
H2WA···H8	2.4900	H35B···O11	2.7200
H2WB···O12^v^	1.94 (4)	H35B···H32	2.5400
			
O1—Rb1—O2	59.93 (4)	C3—C4—H4A	109.00
O1—Rb1—O3	45.03 (4)	C3—C4—H4B	110.00
O1—Rb1—O4	95.56 (4)	H4B—C4—H4C	110.00
O1—Rb1—O5	152.91 (4)	H4A—C4—H4C	109.00
O1—Rb1—O6	140.48 (4)	H4A—C4—H4B	109.00
O1—Rb1—O8	78.10 (5)	C6—C5—H5A	109.00
O2—Rb1—O3	55.22 (4)	C6—C5—H5B	109.00
O2—Rb1—O4	103.34 (5)	H5A—C5—H5B	108.00
O2—Rb1—O5	111.79 (5)	C3—C5—H5B	109.00
O2—Rb1—O6	148.46 (5)	C3—C5—H5A	109.00
O2—Rb1—O8	134.35 (5)	C8—C6—H6	108.00
O3—Rb1—O4	58.49 (4)	C5—C6—H6	109.00
O3—Rb1—O5	108.17 (4)	C7—C6—H6	108.00
O3—Rb1—O6	154.75 (4)	C6—C7—H7C	110.00
O3—Rb1—O8	107.04 (5)	H7A—C7—H7B	109.00
O4—Rb1—O5	59.81 (4)	H7A—C7—H7C	109.00
O4—Rb1—O6	98.18 (4)	H7B—C7—H7C	109.00
O4—Rb1—O8	97.42 (5)	C6—C7—H7A	109.00
O5—Rb1—O6	60.76 (4)	C6—C7—H7B	109.00
O5—Rb1—O8	113.81 (5)	O3—C8—H8	109.00
O6—Rb1—O8	63.51 (5)	C9—C8—H8	109.00
Rb1—O1—C1	88.63 (11)	C6—C8—H8	109.00
Rb1—O2—C2	112.46 (13)	C8—C9—H9	109.00
Rb1—O3—C1	97.92 (11)	C10—C9—H9	109.00
Rb1—O3—C8	99.06 (11)	O4—C9—H9	109.00
C1—O3—C8	115.82 (15)	C11—C10—H10A	111.00
Rb1—O4—C9	117.48 (12)	C9—C10—H10A	111.00
Rb1—O4—C13	99.94 (11)	C9—C10—H10B	111.00
C9—O4—C13	109.45 (16)	H10A—C10—H10B	109.00
Rb1—O5—C14	116.62 (12)	C11—C10—H10B	111.00
Rb1—O5—C19	115.14 (12)	C12—C11—H11	110.00
C14—O5—C19	107.17 (16)	C10—C11—H11	110.00
Rb1—O6—C20	111.96 (12)	C13—C11—H11	110.00
Rb1—O6—C24	132.29 (12)	C11—C12—H12B	109.00
C20—O6—C24	110.54 (16)	C11—C12—H12A	109.00
C24—O7—C29	115.47 (16)	H12A—C12—H12C	109.00
Rb1—O8—C26	133.23 (13)	H12B—C12—H12C	110.00
C32—O9—C33	114.49 (17)	H12A—C12—H12B	110.00
C1—O1—H1O	113.6 (19)	C11—C12—H12C	109.00
Rb1—O1—H1O	70 (2)	C11—C13—H13	107.00
Rb1—O2—H2O	83 (2)	O4—C13—H13	107.00
C2—O2—H2O	107 (2)	C14—C13—H13	107.00
Rb1—O8—H8O	108 (2)	C14—C15—H15B	108.00
C26—O8—H8O	111 (2)	C14—C15—H15A	108.00
H1WA—O12—H1WB	101 (4)	H15A—C15—H15B	107.00
H2WA—O13—H2WB	110 (3)	C16—C15—H15A	108.00
O3—C1—C3	109.42 (17)	C16—C15—H15B	108.00
O3—C1—C2	104.85 (16)	C15—C16—H16A	109.00
O1—C1—C3	108.75 (16)	H16A—C16—H16B	110.00
O1—C1—C2	112.46 (17)	C15—C16—H16B	110.00
O1—C1—O3	110.22 (17)	C15—C16—H16C	109.00
C2—C1—C3	111.08 (18)	H16B—C16—H16C	109.00
O2—C2—C1	114.03 (19)	H16A—C16—H16C	109.00
C1—C3—C4	113.63 (19)	C14—C17—H17B	111.00
C4—C3—C5	112.44 (19)	C18—C17—H17A	111.00
C1—C3—C5	109.18 (18)	H17A—C17—H17B	109.00
C3—C5—C6	112.28 (18)	C18—C17—H17B	111.00
C7—C6—C8	111.80 (19)	C14—C17—H17A	111.00
C5—C6—C7	110.2 (2)	C17—C18—H18A	111.00
C5—C6—C8	109.33 (18)	C19—C18—H18B	111.00
O3—C8—C9	106.52 (16)	H18A—C18—H18B	109.00
C6—C8—C9	112.96 (17)	C17—C18—H18B	111.00
O3—C8—C6	111.44 (18)	C19—C18—H18A	111.00
O4—C9—C8	109.72 (17)	C20—C19—H19	108.00
O4—C9—C10	105.49 (17)	O5—C19—H19	108.00
C8—C9—C10	115.6 (2)	C18—C19—H19	108.00
C9—C10—C11	103.44 (19)	C20—C21—H21C	109.00
C10—C11—C13	99.37 (18)	H21A—C21—H21B	109.00
C10—C11—C12	111.5 (2)	H21A—C21—H21C	109.00
C12—C11—C13	116.5 (2)	H21B—C21—H21C	109.00
O4—C13—C14	108.49 (17)	C20—C21—H21A	110.00
C11—C13—C14	120.96 (19)	C20—C21—H21B	110.00
O4—C13—C11	104.46 (17)	C20—C22—H22A	110.00
O5—C14—C15	108.20 (18)	C23—C22—H22B	110.00
C13—C14—C17	115.12 (19)	C20—C22—H22B	110.00
O5—C14—C13	104.67 (17)	C23—C22—H22A	110.00
C13—C14—C15	111.30 (18)	H22A—C22—H22B	109.00
C15—C14—C17	112.1 (2)	C22—C23—H23B	110.00
O5—C14—C17	104.72 (17)	C24—C23—H23A	110.00
C14—C15—C16	115.5 (2)	H23A—C23—H23B	109.00
C14—C17—C18	105.5 (2)	C24—C23—H23B	110.00
C17—C18—C19	103.20 (19)	C22—C23—H23A	110.00
O5—C19—C18	103.80 (18)	C24—C25—H25A	109.00
O5—C19—C20	110.33 (18)	C26—C25—H25B	109.00
C18—C19—C20	118.20 (19)	H25A—C25—H25B	108.00
C19—C20—C22	111.2 (2)	C24—C25—H25B	109.00
O6—C20—C19	107.86 (17)	C26—C25—H25A	109.00
C19—C20—C21	110.60 (19)	O8—C26—H26	108.00
C21—C20—C22	112.2 (2)	C25—C26—H26	109.00
O6—C20—C21	109.7 (2)	C27—C26—H26	108.00
O6—C20—C22	104.97 (18)	C26—C27—H27	109.00
C20—C22—C23	106.5 (2)	C29—C27—H27	109.00
C22—C23—C24	106.8 (2)	C28—C27—H27	109.00
O7—C24—C25	110.98 (17)	C27—C28—H28B	110.00
C23—C24—C25	113.54 (19)	C27—C28—H28A	110.00
O6—C24—C23	105.60 (17)	H28A—C28—H28C	109.00
O6—C24—C25	108.96 (18)	C27—C28—H28C	109.00
O7—C24—C23	106.67 (18)	H28A—C28—H28B	109.00
O6—C24—O7	110.98 (17)	H28B—C28—H28C	109.00
C24—C25—C26	113.74 (19)	C30—C29—H29	109.00
O8—C26—C25	108.75 (18)	O7—C29—H29	109.00
C25—C26—C27	110.84 (18)	C27—C29—H29	109.00
O8—C26—C27	111.79 (18)	C31—C30—H30	106.00
C28—C27—C29	112.03 (17)	C29—C30—H30	106.00
C26—C27—C29	108.63 (17)	C32—C30—H30	106.00
C26—C27—C28	110.36 (18)	C30—C31—H31B	109.00
O7—C29—C27	109.49 (16)	C30—C31—H31A	109.00
O7—C29—C30	105.14 (16)	H31A—C31—H31C	110.00
C27—C29—C30	115.45 (16)	H31B—C31—H31C	109.00
C29—C30—C31	111.19 (17)	C30—C31—H31C	109.00
C31—C30—C32	111.26 (18)	H31A—C31—H31B	110.00
C29—C30—C32	115.42 (17)	C34—C32—H32	109.00
O9—C32—C34	104.64 (17)	O9—C32—H32	109.00
O9—C32—C30	107.34 (17)	C30—C32—H32	109.00
C30—C32—C34	117.66 (17)	O9—C33—H33C	109.00
C32—C34—C36	115.07 (19)	H33A—C33—H33B	109.00
C32—C34—C35	110.4 (2)	H33A—C33—H33C	110.00
C35—C34—C36	107.26 (19)	H33B—C33—H33C	109.00
O11—C36—C34	118.9 (2)	O9—C33—H33B	109.00
O10—C36—C34	116.7 (2)	O9—C33—H33A	109.00
O10—C36—O11	124.3 (2)	C35—C34—H34	108.00
O2—C2—H2A	109.00	C32—C34—H34	108.00
C1—C2—H2B	109.00	C36—C34—H34	108.00
O2—C2—H2B	109.00	C34—C35—H35B	109.00
C1—C2—H2A	109.00	C34—C35—H35C	110.00
H2A—C2—H2B	108.00	H35A—C35—H35C	109.00
C4—C3—H3	107.00	H35B—C35—H35C	109.00
C5—C3—H3	107.00	H35A—C35—H35B	109.00
C1—C3—H3	107.00	C34—C35—H35A	110.00
C3—C4—H4C	109.00		
			
O2—Rb1—O1—C1	45.16 (11)	C24—O6—C20—C21	−96.4 (2)
O3—Rb1—O1—C1	−24.16 (10)	Rb1—O6—C20—C22	−177.83 (16)
O4—Rb1—O1—C1	−57.05 (11)	C20—O6—C24—C25	−144.76 (17)
O5—Rb1—O1—C1	−33.88 (16)	Rb1—O6—C20—C19	−59.13 (18)
O6—Rb1—O1—C1	−167.10 (10)	Rb1—O6—C24—C23	−174.19 (13)
O8—Rb1—O1—C1	−153.49 (12)	Rb1—O6—C24—C25	63.5 (2)
O1—Rb1—O2—C2	−19.70 (12)	C20—O6—C24—C23	−22.5 (2)
O3—Rb1—O2—C2	33.98 (12)	C29—O7—C24—O6	66.0 (2)
O4—Rb1—O2—C2	69.02 (14)	C24—O7—C29—C30	−173.25 (16)
O5—Rb1—O2—C2	131.52 (13)	C29—O7—C24—C25	−55.3 (2)
O6—Rb1—O2—C2	−159.23 (12)	C29—O7—C24—C23	−179.48 (16)
O8—Rb1—O2—C2	−45.65 (16)	C24—O7—C29—C27	62.1 (2)
O1—Rb1—O3—C1	24.15 (10)	Rb1—O8—C26—C27	58.1 (2)
O2—Rb1—O3—C1	−56.15 (10)	Rb1—O8—C26—C25	−64.6 (2)
O4—Rb1—O3—C1	164.80 (12)	C33—O9—C32—C30	−88.8 (2)
O5—Rb1—O3—C1	−160.49 (10)	C33—O9—C32—C34	145.5 (2)
O6—Rb1—O3—C1	140.13 (12)	O3—C1—C2—O2	−49.1 (2)
O8—Rb1—O3—C1	76.49 (11)	O1—C1—C3—C5	−64.8 (2)
O1—Rb1—O3—C8	−93.72 (12)	O1—C1—C2—O2	70.7 (2)
O2—Rb1—O3—C8	−174.02 (13)	O3—C1—C3—C4	−177.94 (18)
O4—Rb1—O3—C8	46.93 (11)	C2—C1—C3—C5	170.96 (17)
O5—Rb1—O3—C8	81.64 (12)	C3—C1—C2—O2	−167.20 (17)
O6—Rb1—O3—C8	22.26 (17)	O3—C1—C3—C5	55.7 (2)
O8—Rb1—O3—C8	−41.38 (12)	O1—C1—C3—C4	61.6 (2)
O1—Rb1—O4—C9	7.54 (13)	C2—C1—C3—C4	−62.7 (2)
O2—Rb1—O4—C9	−52.83 (13)	C1—C3—C5—C6	−55.6 (2)
O3—Rb1—O4—C9	−19.25 (12)	C4—C3—C5—C6	177.33 (19)
O5—Rb1—O4—C9	−160.49 (14)	C3—C5—C6—C8	53.2 (2)
O6—Rb1—O4—C9	150.39 (13)	C3—C5—C6—C7	176.45 (19)
O8—Rb1—O4—C9	86.23 (13)	C7—C6—C8—O3	−174.11 (19)
O1—Rb1—O4—C13	125.72 (12)	C7—C6—C8—C9	66.0 (2)
O2—Rb1—O4—C13	65.35 (12)	C5—C6—C8—C9	−171.68 (18)
O3—Rb1—O4—C13	98.93 (12)	C5—C6—C8—O3	−51.8 (2)
O5—Rb1—O4—C13	−42.32 (11)	O3—C8—C9—O4	61.7 (2)
O6—Rb1—O4—C13	−91.43 (12)	C6—C8—C9—O4	−175.61 (17)
O8—Rb1—O4—C13	−155.60 (12)	O3—C8—C9—C10	−57.4 (2)
O1—Rb1—O5—C14	−15.42 (18)	C6—C8—C9—C10	65.3 (2)
O2—Rb1—O5—C14	−81.62 (13)	O4—C9—C10—C11	24.3 (2)
O3—Rb1—O5—C14	−22.65 (14)	C8—C9—C10—C11	145.68 (19)
O4—Rb1—O5—C14	11.52 (12)	C9—C10—C11—C12	84.7 (2)
O6—Rb1—O5—C14	132.48 (14)	C9—C10—C11—C13	−38.7 (2)
O8—Rb1—O5—C14	96.16 (13)	C12—C11—C13—O4	−79.4 (2)
O1—Rb1—O5—C19	−142.30 (13)	C12—C11—C13—C14	43.1 (3)
O2—Rb1—O5—C19	151.50 (13)	C10—C11—C13—O4	40.4 (2)
O3—Rb1—O5—C19	−149.52 (13)	C10—C11—C13—C14	162.8 (2)
O4—Rb1—O5—C19	−115.35 (14)	C11—C13—C14—O5	170.54 (18)
O6—Rb1—O5—C19	5.60 (13)	O4—C13—C14—C17	45.4 (2)
O8—Rb1—O5—C19	−30.71 (15)	O4—C13—C14—C15	174.40 (18)
O1—Rb1—O6—C20	−174.06 (11)	O4—C13—C14—O5	−69.0 (2)
O2—Rb1—O6—C20	−56.05 (16)	C11—C13—C14—C17	−75.1 (3)
O3—Rb1—O6—C20	97.85 (15)	C11—C13—C14—C15	53.9 (3)
O4—Rb1—O6—C20	76.78 (13)	O5—C14—C15—C16	−52.7 (3)
O5—Rb1—O6—C20	28.29 (12)	C17—C14—C15—C16	−167.7 (2)
O8—Rb1—O6—C20	171.03 (14)	C13—C14—C15—C16	61.8 (3)
O1—Rb1—O6—C24	−22.6 (2)	O5—C14—C17—C18	−6.7 (2)
O2—Rb1—O6—C24	95.38 (18)	C15—C14—C17—C18	110.4 (2)
O3—Rb1—O6—C24	−110.73 (18)	C13—C14—C17—C18	−121.0 (2)
O4—Rb1—O6—C24	−131.80 (16)	C14—C17—C18—C19	−16.7 (2)
O5—Rb1—O6—C24	179.71 (18)	C17—C18—C19—C20	157.2 (2)
O8—Rb1—O6—C24	−37.55 (16)	C17—C18—C19—O5	34.7 (2)
O1—Rb1—O8—C26	−131.8 (2)	O5—C19—C20—O6	64.6 (2)
O2—Rb1—O8—C26	−109.08 (19)	O5—C19—C20—C21	−55.4 (3)
O3—Rb1—O8—C26	−166.75 (18)	C18—C19—C20—C21	−174.5 (2)
O4—Rb1—O8—C26	134.01 (19)	C18—C19—C20—C22	60.1 (3)
O5—Rb1—O8—C26	73.8 (2)	C18—C19—C20—O6	−54.6 (3)
O6—Rb1—O8—C26	38.53 (18)	O5—C19—C20—C22	179.2 (2)
Rb1—O1—C1—O3	39.06 (14)	C21—C20—C22—C23	102.9 (3)
Rb1—O1—C1—C2	−77.54 (16)	C19—C20—C22—C23	−132.6 (2)
Rb1—O1—C1—C3	159.00 (15)	O6—C20—C22—C23	−16.2 (3)
Rb1—O2—C2—C1	−8.4 (2)	C20—C22—C23—C24	3.2 (3)
C8—O3—C1—O1	60.8 (2)	C22—C23—C24—C25	130.5 (2)
Rb1—O3—C1—C2	77.91 (14)	C22—C23—C24—O6	11.2 (3)
C8—O3—C1—C2	−177.97 (16)	C22—C23—C24—O7	−107.0 (2)
Rb1—O3—C1—C3	−162.87 (13)	C23—C24—C25—C26	167.94 (19)
C8—O3—C1—C3	−58.8 (2)	O6—C24—C25—C26	−74.7 (2)
Rb1—O3—C8—C6	160.65 (14)	O7—C24—C25—C26	47.8 (3)
C1—O3—C8—C6	57.2 (2)	C24—C25—C26—O8	74.8 (2)
Rb1—O3—C1—O1	−43.34 (16)	C24—C25—C26—C27	−48.5 (3)
C1—O3—C8—C9	−179.16 (16)	O8—C26—C27—C28	168.45 (18)
Rb1—O3—C8—C9	−75.74 (15)	O8—C26—C27—C29	−68.4 (2)
C9—O4—C13—C11	−27.0 (2)	C25—C26—C27—C28	−70.1 (2)
Rb1—O4—C13—C11	−150.96 (14)	C25—C26—C27—C29	53.1 (2)
C13—O4—C9—C10	1.7 (2)	C28—C27—C29—C30	−54.8 (2)
Rb1—O4—C9—C8	−10.6 (2)	C28—C27—C29—O7	63.6 (2)
C13—O4—C9—C8	−123.50 (18)	C26—C27—C29—C30	−176.98 (18)
C9—O4—C13—C14	−157.26 (17)	C26—C27—C29—O7	−58.6 (2)
Rb1—O4—C13—C14	78.78 (16)	O7—C29—C30—C31	54.8 (2)
Rb1—O4—C9—C10	114.64 (15)	C27—C29—C30—C32	−56.5 (2)
C14—O5—C19—C18	−40.8 (2)	O7—C29—C30—C32	−177.30 (16)
Rb1—O5—C19—C18	90.72 (16)	C27—C29—C30—C31	175.53 (18)
Rb1—O5—C19—C20	−36.9 (2)	C29—C30—C32—O9	159.25 (16)
Rb1—O5—C14—C17	−101.14 (16)	C31—C30—C32—C34	44.7 (2)
Rb1—O5—C14—C13	20.36 (19)	C29—C30—C32—C34	−83.2 (2)
C19—O5—C14—C17	29.6 (2)	C31—C30—C32—O9	−72.9 (2)
C19—O5—C14—C13	151.08 (17)	O9—C32—C34—C35	−59.7 (2)
Rb1—O5—C14—C15	139.13 (15)	O9—C32—C34—C36	178.77 (18)
C19—O5—C14—C15	−90.2 (2)	C30—C32—C34—C35	−178.69 (18)
C14—O5—C19—C20	−168.41 (17)	C30—C32—C34—C36	59.8 (3)
Rb1—O6—C24—O7	−59.0 (2)	C32—C34—C36—O10	−127.2 (3)
C20—O6—C24—O7	92.8 (2)	C32—C34—C36—O11	54.9 (3)
C24—O6—C20—C22	24.4 (2)	C35—C34—C36—O10	109.6 (3)
C24—O6—C20—C19	143.07 (18)	C35—C34—C36—O11	−68.3 (3)
Rb1—O6—C20—C21	61.40 (19)		

Symmetry codes: (i) −*x*+2, *y*−1/2, −*z*+3/2; (ii) −*x*+2, *y*+1/2, −*z*+3/2; (iii) −*x*+1, *y*−1/2, −*z*+3/2; (iv) *x*−1/2, −*y*+3/2, −*z*+2; (v) −*x*+1, *y*+1/2, −*z*+3/2; (vi) −*x*+3/2, −*y*+1, *z*−1/2; (vii) *x*−1, *y*, *z*; (viii) *x*+1/2, −*y*+3/2, −*z*+2; (ix) *x*+1, *y*, *z*; (x) −*x*+3/2, −*y*+1, *z*+1/2.

### Hydrogen-bond geometry (Å, °)

**Table:**

*D*—H···*A*	*D*—H	H···*A*	*D*···*A*	*D*—H···*A*
O12—H1WA···O9^i^	0.78 (4)	2.20 (4)	2.959 (3)	167 (3)
O1—H1O···O11	0.86 (4)	1.82 (4)	2.651 (2)	162 (3)
O12—H1WB···O2	0.71 (3)	2.06 (3)	2.743 (3)	162 (4)
O2—H2O···O10	0.82 (4)	1.74 (4)	2.538 (3)	162 (3)
O13—H2WA···O1	0.92 (3)	1.93 (3)	2.807 (3)	158 (3)
O13—H2WB···O12^v^	0.83 (4)	1.94 (4)	2.762 (3)	174 (3)
O8—H8O···O13	0.70 (3)	2.04 (3)	2.723 (3)	168 (3)
C10—H10B···O3	0.97	2.52	2.920 (3)	104
C17—H17A···O4	0.97	2.46	2.847 (3)	104
C18—H18A···O6	0.97	2.58	2.963 (3)	103
C21—H21B···O5	0.96	2.53	2.869 (3)	101
C29—H29···O6	0.98	2.60	2.924 (3)	100
C31—H31C···O7	0.96	2.44	2.787 (3)	101
C35—H35A···O9	0.96	2.43	2.813 (3)	103

Symmetry codes: (i) −*x*+2, *y*−1/2, −*z*+3/2; (v) −*x*+1, *y*+1/2, −*z*+3/2.
